# Optimizing Oncology Generic Medication Selection in the Gulf Region: Expert Consensus and MCDA Tool Development

**DOI:** 10.36469/001c.140955

**Published:** 2025-07-18

**Authors:** Anas Hamad, Omar AbdulAziz, Osama Abu Tabar, Sara Al Balushi, Sana Alblooshi, Mohamad Alfar, Eren Dawoud, Fahmeeda Khan, Abdulrahman Nabeel, Wael Sayam, Ahmad ElSheashaey

**Affiliations:** 1 Pharmacy Department National Center for Cancer Care & Research, Hamad Medical Corporation, Doha, Qatar; 2 College of Pharmacy, QU Health Sector, Qatar University, Doha, Qatar; 3 Pharmacy Department King Hamad University Hospital, Royal Medical Services, Busaiteen, Bahrain; 4 Pharmacy Department Fatima Bint Mubarak Center, Cleveland Clinic Abu Dhabi, UAE; 5 Pharmaceutical Care Directorate Directorate General of Medical Supplies, Ministry of Health, Muscat, Oman; 6 Pharmacy Department Tawam Hospital, College of Medicine and Health Sciences, Al Ain, UAE; 7 College of Medicine and Health Sciences, United Arab Emirates University, Al Ain, UAE; 8 Pharmacy Department Burjeel Cancer Institute, Burjeel Medical City, Abu Dhabi, UAE; 9 Medical Affairs, Hikma Pharmaceuticals, Dubai, UAE; 10 Pharmacy Department Mohammed Bin Khalifa Bin Salman AlKhalifa Cardiac Center, Awali, Bahrain; 11 Pharmacy Department Salmanyia Medical Complex, Manama, Bahrain; 12 Pharmacy Department Kuwait Cancer Control Center, Shuwaikh, Kuwait

**Keywords:** Gulf Cooperation Council, oncology genetics, multicriteria decision analysis, regulatory frameworks, manufacturing quality, supply reliability, pharmacovigilance, healthcare accessibility

## Abstract

**Background:** Cancer remains a leading global health challenge, with high mortality rates and increasing financial burdens on healthcare systems. In the Gulf Cooperation Council countries, generic oncology medications offer a cost-effective alternative to patented treatments. However, disparities in regulatory frameworks and concerns about quality and supply reliability hinder their widespread adoption. Addressing these challenges is crucial to ensuring optimal access to oncology treatments. **Methods:** This study employed multicriteria decision analysis to evaluate key factors influencing the selection of off-patent oncology drugs. A structured survey was conducted among 10 formulary management experts from the United Arab Emirates, Qatar, Kuwait, Bahrain, and Oman. Participants engaged in a 3-hour workshop to assess and prioritize critical decision-making criteria through guided discussions, case studies, and weighting surveys. **Results:** The analysis identified manufacturing quality (30.8%), cost (20%), and use in reference countries (14.6%) as the most critical factors in selecting generic oncology mediations. Supply reliability (13%), regulatory aspects (8%), and pharmacovigilance services and extra services (6.8% each) also played supporting roles. The structured evaluation framework developed through this study provides insights into the factors shaping formulary decisions in the Gulf Cooperation Council region and highlights areas requiring regulatory and logistical improvements. **Conclusion:** This expert consensus underscores the need for a balanced approach that ensures quality, affordability, and accessibility in adopting oncology generics. Strengthening regulatory frameworks, improving pharmacovigilance, and enhancing stakeholder education are essential steps to optimizing the integration of oncology drugs into regional healthcare systems. These findings provide a foundation for policy recommendations aimed at improving generic drug adoption and patient outcomes in oncology care.

## INTRODUCTION

Cancer remains a leading global health challenge, causing nearly 10 million deaths annually.^1^ In the United Arab Emirates (UAE), it is the third leading cause of death,^2^ with breast, colorectal, and lung cancers being the most prevalent.^3^ The increasing cancer burden places immense pressure on healthcare systems, driven by the high costs of innovative treatments and systemic inefficiencies in many countries.^4–7^

To mitigate these challenges, generic drugs have emerged as cost-effective alternatives to patented medications. Generics contain the same active ingredients and demonstrate similar bioavailability, offering comparable therapeutic efficacy at a lower cost. As patents for more oncology drugs expire, the availability of generics is expanding, making the continuous evaluation of their quality, safety, and efficacy essential.^8^ However, heterogeneity in regulatory frameworks for generic drug approval across countries creates inconsistencies, and causes delays in access to essential treatments.^9^ In particular, Arabian Gulf nations face disparities in standardized approval mechanisms, reimbursement evaluation, and procurement strategies of pharmaceuticals, raising concerns about the consistency and reliability of generic oncology treatments.^10,11^

Multi-criteria decision analysis (MCDA) provides a structured approach to complex healthcare decision-making, integrating multiple factors such as cost, clinical effectiveness, safety, and patient quality of life.^12^ This paper explores the potential of MCDA in assessing oncology generics within the Gulf region, emphasizing its role in improving regulatory processes and ensuring optimal treatment outcomes.

## METHODS

A multidisciplinary panel of 10 formulary management experts (including pharmacists, health economists, and procurement specialists) from the UAE, Qatar, Kuwait, Bahrain, and Oman participated in a structured workshop held on October 29, 2024, in Dubai. Participants were selected based on their extensive experience (minimum 10 years) in oncology formulary management, participation in pharmacy and therapeutics committees, and involvement in national healthcare projects. The objective was to identify optimization strategies for selecting off-patent oncology medications and develop an MCDA tool for value assessment. To enhance rigor, the process involved 3 phases: (1) a pre-workshop survey (Round 1) where experts independently weighted key criteria (eg, manufacturing quality, cost, supply reliability); (2) a 3-hour workshop with case studies and discussions to refine interpretations and address discrepancies; and (3) a post-discussion anonymous voting round (Round 2) to finalize weights, ensuring consensus and mitigating bias. The direct percentage allocation method was used for weighting, with the sum of all criteria fixed at 100%. While no formal sensitivity analysis was conducted, the iterative pre/post-discussion voting improved weight stability. Questions were shared beforehand, and all experts had long-standing roles in hemato-oncology care, procurement, and formulary committees, incorporating cross-functional insights within their institutions. Industry sponsorship was disclosed, but weightings were determined independently by the panel. **Table 1** summarizes the key discussion topics covered during the meeting.

Table 1. **Key Discussion Topics**

1. What are your experiences with using high-quality generic medications for oncology patients in the lower GCC region?
2. What is the role of high-quality generics in cost containment?
3. In your opinion, are lower GCC patients generally receptive to the use of generic medications for their oncology treatments? How do you manage any feedback they might have?
4. What are the challenges you face in terms of availability and access to medications which improved by high-quality generics?
5. How do you evaluate the cost-effectiveness of new oncology medications when considering formulary addition?
6. What is the process for assessing the budget impact of adding a new oncology drug to the formulary, and what factors are most critical?
7. How do you involve key stakeholders (physicians, payers, patients) in the decision-making process for formulary additions?
8. Most essential quality measures for assessing off-patent oncology medications:
  - Manufacturing quality
  - Cost
  - Supply reliability
  - Use in reference countries
  - Regulatory aspects
  - Provision of pharmacovigilance services
  - Extra services
9. What criteria do you consider most important in the MCDA for off-patent cancer medications?
10. How does the outcome of your MCDA influence clinical practice and treatment protocols in your institution?
11. How do you balance cost savings from off-patent medications with potential differences in efficacy or safety compared to branded alternatives? (Insurance parameter)
12. What are your recommendations for pharma companies for promoting healthcare education in GCC?

Abbreviations: GCC, Gulf Cooperation Council; MCDA, multi-criteria decision analysis.

## RESULTS

### Optimizing Oncology Generic Medication Selection in the Gulf Region

The following areas were generated from the panel discussion and considered essential for optimizing generic medication selection in the Gulf Region.

**Generics in oncology:** Generic medications have become a cornerstone of oncology, providing cost reduction and improved access to lifesaving treatments. Their introduction has significantly lowered treatment expenses, often aligning with insurance requirements and overcoming barriers such as high costs, restrictive policies, patient-specific factors, healthcare facility limitations, and delays in healthcare provider decisions. Additionally, generics contribute to sustainability by ensuring a stable medication supply.

**Market introduction and approval processes:** For a new generic drug to be successfully introduced to the market, it must demonstrate clinical effectiveness, address medical needs, enhance patient satisfaction, and be cost-effective. A viable and sustainable generic must ensure timely access for the appropriate patients across different disease stages while maintaining affordability and efficacy throughout the prescribed treatment duration. Several factors influence generic adoption including quality, convenience, and service elements such as patient support programs and pharmacovigilance.

The approval process in Oman serves as an example of structured formulary management, involving three key committees: the pharmacy and therapeutics committee in hospitals for clinical approval, the task force committee which is a subcommittee who works under the central drug committee to review all application forms for their eligibility to be included to the formulary, and the central drug committee chaired by the under secretary of health affairs for final approval and inclusion of medicines in ministry of health formulary.

This process starts with a physician’s request, which is evaluated at the pharmacy and therapeutic committee within the hospitals, reviewed by the task forces committee and once approved by the central drug committee, the medication is procured and distributed for use in the healthcare system within the ministry of health.

Despite its structured approach, pharmacoeconomics faces several challenges, including the rising costs of advanced therapies such as targeted therapies and biologics, limited financial resources, and rapidly evolving treatment guidelines. Effective formulary management must balance evidence-based decisions, affordability, patient demand, and institutional priorities. The lack of a structured framework for evaluating clinical outcomes and drug utilization further complicates the process. To address these gaps, Oman has developed a health technology assessment (HTA) roadmap outlining decision criteria and guidelines, while also introducing managed entry agreements, initially for some rare diseases, though not yet for oncology treatments.

**Challenges of adopting generics in health care**: While generics play a crucial role in cost-effective cancer treatment, several challenges hinder their widespread adoption. Experts raised concerns regarding the quality and acceptance of generics, using the example of an imatinib, which has shown efficacy but requires stricter quality controls due to observed variability in sarcoma treatment and hypersensitivity reactions.

Supply chain disruptions (eg, doxorubicin, irinotecan shortages) and lack of pharmacovigilance systems across the Gulf Cooperation Council (GCC) further complicate adoption. Patients often resist switching due to familiarity with originator packaging, though provider counseling can mitigate this. Limited local manufacturer representation exacerbates supply issues, highlighting a need for regional production standards.

From a patient’s perspective, reluctance to switch to generics is common, often driven by familiarity with medication packaging and prior experiences. Counseling and reassurance from healthcare providers have proven effective in easing patient concerns. Additionally, the limited registration and availability of high-quality generics in the GCC restrict options for both patients and physicians. Supply chain challenges are further exacerbated by the absence of local supplier representatives, making it difficult to address issues promptly. Global supply chain disruptions, particularly for oncology medications, add another layer of complexity to the problem.

**Cost considerations and economic impact**: Balancing cost efficiency with maintentance of high-quality care remains a significant challenge. While generics offer significant savings, concerns about their effectiveness and patient preferences can complicate decision making. The affordability of generics allows for larger stockpiles and easier inventory management, something that is often not feasible with patented medications due to their high cost. However, the tender process, which prioritizes securing the lowest prices procurement, can further complicate medication selection.

Cost-effectiveness remains a central issue in pharmacoeconomics. The discussion emphasized the need for patient assessment and treatment comparisons, citing ravulizumab as a cost-effective option due to its less frequent dosing compared with eculizumab, leading to lower costs and greater patient convenience. However, budget constraints often limit access to high-cost medications, especially when survival benefits are marginal This issue is further compounded by the absence of an official cost-effectiveness threshold in the region, making comprehensive economic evaluations difficult.

Cost considerations extend beyond direct expenses to factors like adherence, treatment frequency, and indirect costs such as transportation and healthcare staff expenses. While fixed budgets for generics can help lower per-patient costs, indirect costs related to adverse events and patient safety must also be factored in. In the long run, safer drugs tend to be more cost-effective despite their higher initial price.

**Enhancing healthcare education in the GCC:** While many pharmaceutical companies offer online educational courses for healthcare professionals, the panel emphasized that sharing practical experiences across GCC countries could be more effective. Pharmaceutical companies should collaborate with hospital pharmacy departments to organize awareness campaigns that directly educate patients about medication changes. Similarly, educating healthcare professionals, particularly pharmacists, about generics is crucial. Companies should also support patient and physician education through printed materials to reassure them about the quality and efficacy of generic medications.

**Institutional variability in procurement:** Procurement approaches differ among healthcare institutions. Some private hospitals require oncologist approval before procuring patient-specific medications, while other institutions without structured economic evaluation methods rely on patient requests and cost analysis to determine which medications to include in the formulary. In Qatar, negotiations focus on price while considering the broader impact of medications on patient quality of life, hospital visits, and overall treatment outcomes.

**Lack of patient involvement in formulary decisions:** All attendees agreed that patient advocacy is currently absent from the formulary addition process, and patients are not consulted regarding their medication needs. While Qatar has a cancer society and patient support groups focused on toxicity management and quality of life, there is no formal mechanism for patient involvement in formulary decision-making. Addressing this gap could enhance patient-centered care and improve the acceptance and adherence of new medications in the healthcare system.

### The Role of MCDA

MCDA is emerging as a critical decision-making tool for evaluating off-patent medications, particularly in resource-constrained settings where patient outcomes must be carefully balanced with economic considerations. It allows for the systematic assessment of treatment options based on multiple factors, including effectiveness, cost, safety, quality of life, and equity in access. Effectiveness is measured through survival rates and tumor response, while cost considerations highlight the affordability of generics. Safety evaluations focus on side effects and long-term data, and quality of life is assessed through factors like ease of administration and symptom control. Equity ensures that treatments remain accessible to diverse patient populations.

The panel highlighted several challenges associated with MCDA, including limited data on some off-patent drugs, subjective weighting of criteria, and the need for continuous reassessment due to the rapid evolution of cancer treatments. Despite these challenges, MCDA is becoming an increasingly important alternative to a full HTA, particularly when resources or time are limited. As more cancer medications become off-patent, MCDA helps balance clinical outcomes with healthcare resource allocation, making it a versatile tool for both generics and new originator products.

The discussion explored various factors influencing medication selection and evaluation. Cost-effectiveness plays a central role, balancing affordability with clinical benefits. The extent to which a drug is widely used in reference countries adds to its reliability, while the safety profile is determined by established safety data and post-market surveillance. Regulatory compliance ensures adherence to international and national standards, while supply reliability remains crucial to prevent shortages. Experience in reference countries, additional services from pharmaceutical companies such as support programs, training, and pharmacovigilance initiatives, further contribute to the decision-making process.

The panel emphasized the importance of manufacturing quality and its impact on pharmacovigilance. Unregistered products may receive temporary registration until they meet the criteria for permanent approval. Packaging quality and batch uniformity also influence drug efficacy, reinforcing the need for reliable pharmacovigilance systems. Regulatory status remains a crucial determinant in selecting generics, ensuring compliance with stringent quality and safety standards.

Finally, logistical and clinical considerations, such as the country of origin, manufacturer reputation, and approval status, play a vital role in medication selection. The panel underscored the importance of mandatory inspections and reference country usage as prerequisites for product registration in Oman. This structured approach helps ensure the availability of high-quality generics while maintaining a balance between cost-effectiveness, patient safety, and regulatory integrity.

### MCDA Survey Results and Analysis

**Survey design and findings:** An MCDA survey was conducted among healthcare stakeholders in the GCC region to determine the most critical quality measures for assessing off-patent oncology medications. Ten formulary management experts participated in the survey, assigning percentages to each criterion based on its importance, ensuring the total equaled 100%.

**Figure 1.**
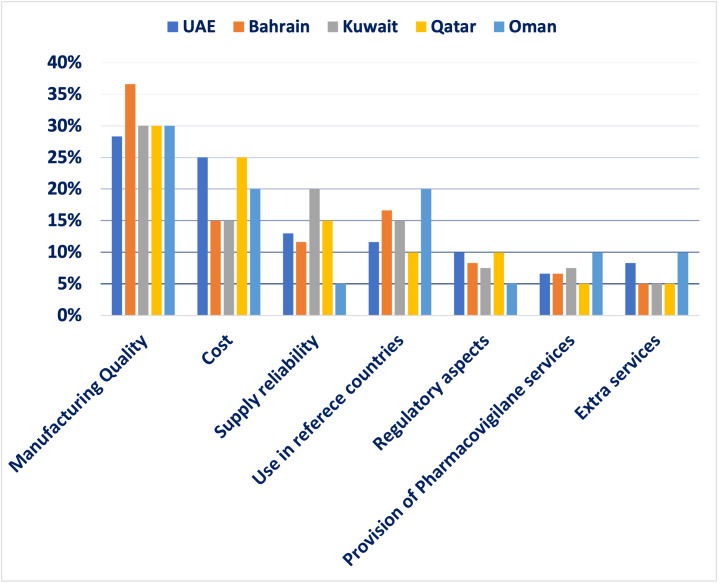
MCDA Survey Findings Abbreviation: MCDA, multi-criteria decision analysis; UAE, United Arab Emirates.

**Figure 2.**
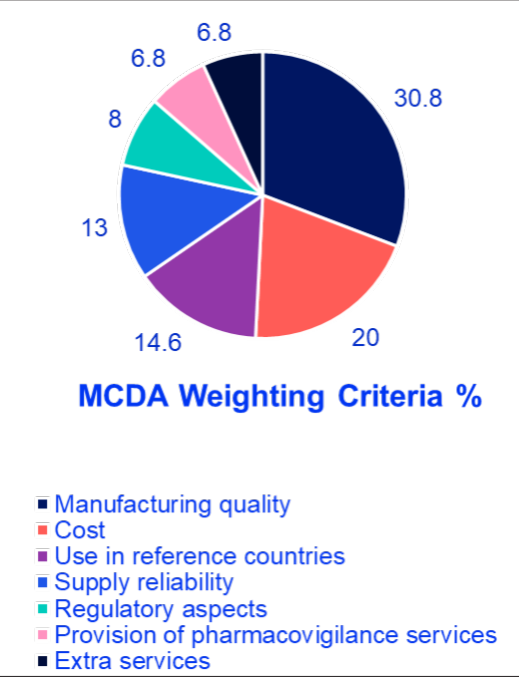
MCDA Weighting Criteria Abbreviation: MCDA, multi-criteria decision analysis.

The findings revealed that manufacturing quality was the most crucial factor, accounting for 30.8% of the total weight, highlighting the region’s focus on safety and efficacy and reflecting demand for international production standards. Cost ranked second at 20%, indicating a significant concern for financial sustainability in oncology treatment. Use in reference countries was the third most influential factor at 14.6%, reflecting reliance on external validation and regulatory approvals in well-established markets. Supply reliability accounted for 13%, underlining the necessity of consistent drug availability to ensure treatment continuity for oncology patients. Regulatory aspects were assigned 8%, demonstrating their importance but ranking lower compared to quality and cost; aligns with Oman’s mandatory inspections. Provision of pharmacovigilance services and extra services each received 6.8%, signifying a relatively lower weight but still relevant considerations in decision making. Finally, extra services received 6.8% showing that they are valuable but not decisive (eg, home delivery programs). These results emphasize a balanced approach in selecting generic oncology medications, where clinical effectiveness, financial considerations, and logistical feasibility are all carefully weighed (**Figure 1 and Figure 2**). Afterward, detailed scoring criteria were developed and agreed upon by the group where each criterion will get a score from 100% to 0% based on several scoring elements. There are 2 scenarios where experts agreed that the product should be excluded without looking into any other criteria; if there is no good manufacturing practices certification for the product whatsoever, or if the product is not approved in any reference market. More details about the scoring tool can be found in **Table 2.**

**Table 2.** MCDA Scoring Criteria for Oncology Generics (Gulf Region)

**Criterion Name**	**Possible Scoring Elements**	**Score (%)**
Manufacturing quality	WHO/EMA/FDA GMP certificates for API and finished product	100
	WHO or EMA/FDA GMP for one only (API or finished product)	70
	National authority GMP only	40
	No GMP certification	EXCLUSION
Cost	Price ≤ 40% of originator	100
	Price 41-60% of originator	70
	Price 61-75% of originator	45
	Price 76-90% of originator	20
	Price >90% of originator	0
Use in reference countries	Product is marketed in ≥3 GCC or ICH reference countries (eg, US, UK, EMA)	100
	Marketed in 1-2 reference countries	75
	Approved in GCC but not in ICH	50
	Not approved in any reference market	EXCLUSION
Supply reliability	No supply issues in past 2 years	100
	Minor delays but >75% reliable	70
	Moderate delays (50-75% reliability)	40
	Frequent shortages or <50% reliability	0
Regulatory aspects	Registered in country of use via full dossier or abridged submission (bioequivalence data)	100
	Registered under fast-track or abridged process (no local bioequivalence)	70
	Temporary approval only	40
	Unregistered	0
Pharmacovigilance	Local reporting system + manufacturer has active PV system	100
	Manufacturer has passive PV system	50
	No evidence of PV system	0
Extra services	Offers HCP training, patient support, and educational materials	100
	Offers HCP training only	50
	No extra services	0

Abbreviations: API, active pharmaceutical ingredient; EMA, European Medicines Agency; FDA, US Food and Drug Administration; GCC, Gulf Cooperation Council; GMP, good manufacturing practices; HCP, healthcare professional; ICH, International Council for Harmonisation of Technical Requirements for Pharmaceuticals for Human Use; PV, pharmacovigilance; WHO, World Health Organization.

**Analysis of findings:** The survey findings suggest that healthcare decision-makers in the GCC prioritize a combination of quality assurance, affordability, and supply reliability when selecting off-patent oncology medications. The emphasis on manufacturing quality highlights the demand for high production standards, stringent quality control, and proven clinical effectiveness. Cost considerations ranked highly, reflecting the need to optimize healthcare spending without compromising patient outcomes. The importance of supply reliability underscores the necessity of uninterrupted medication access, particularly in oncology, where treatment delays can significantly impact survival rates. The use of generics in reference countries suggests a preference for medications already approved in internationally recognized regulatory systems, reinforcing trust in product efficacy and safety. Regulatory aspects, pharmacovigilance services, and extra services play supporting roles in ensuring compliance and patient safety but are considered secondary to manufacturing quality and cost.

The MCDA survey findings offer valuable insights for policymakers, healthcare providers, and pharmaceutical companies operating in the GCC. Ensuring high-quality, cost-effective, and reliably available oncology medications remains a top priority. Addressing logistical challenges and strengthening regulatory frameworks will further support informed decision-making in the procurement and use of off-patent oncology medications.

## Key Recommendations of the Expert Panel

### Regional level

1. Develop standardized guidelines for oncology generics across GCC countries, aligned with international regulatory agencies (eg, EMA, FDA, WHO).
2. Establish a regional pharmacovigilance system to monitor adverse drug reactions and enhance patient safety.
3. Strengthen international collaborations to secure stable supply chains, ensuring consistent access to critical oncology medications.
4. Encourage participation in global drug evaluation networks to facilitate access to real-world evidence on oncology generics.
5. Implement international cost-effectiveness frameworks to support evidence-based decision-making on generic adoption.

### National level

1. Establish country-specific evaluation processes while aligning with GCC-wide approval mechanisms for oncology generics.
2. Launch awareness campaigns to improve acceptance and confidence in generic medications among patients and physicians.
3. Incorporate MCDA in national procurement policies to balance cost and quality considerations.
4. Encourage domestic production of high-quality generics to reduce dependency on international suppliers and minimize shortages.
5. Implement risk-sharing agreements between healthcare systems and pharmaceutical companies to facilitate cost-effective generic adoption while ensuring performance-based drug selection.

By adopting these recommendations, healthcare systems can enhance access to affordable, high-quality oncology treatments, ultimately improving patient outcomes and healthcare sustainability.

### Limitations

This study has several limitations that warrant consideration. The findings are based on a single, 3-hour workshop involving a small, homogeneous sample of formulary managers, which may introduce selection bias and underrepresent key stakeholder perspectives—such as oncologists and patients. The absence of a fully structured Delphi technique or iterative consensus mechanism (eg, multiple rounds of feedback) may raise questions about stability and robustness of the derived criterion weights. Additionally, the lack of real-world validation—either retrospective or prospective—leaves the tool’s practical utility and impact on formulary decisions uncertain. These limitations may limit the generalizability of the results. Given these constraints, policy recommendations and broader implementation need further refinement, Delphi rounds, and pilot testing in real-world settings.

## CONCLUSION

The study underscores the importance of manufacturing quality, cost, and regulatory alignment in the selection of generic oncology medications in the GCC. While cost savings drive the adoption of generics, ensuring product quality, supply reliability, and patient trust remains paramount. The reliance on international regulatory approvals emphasizes the need for harmonized evaluation processes within the region. Pharmacovigilance and supply chain management must be strengthened to mitigate quality concerns and prevent shortages.

Furthermore, patient and provider engagement in formulary decisions remains limited, highlighting the need for structured educational programs and transparent communication to improve acceptance. The findings support the integration of MCDA as a systematic approach to decision-making, enabling stakeholders to balance clinical efficacy, economic feasibility, and regulatory requirements.

Addressing logistical barriers, enhancing local regulatory frameworks, and fostering collaboration between healthcare institutions and pharmaceutical companies will improve the accessibility and reliability of oncology generics. Policymakers should leverage these insights to develop sustainable, high-quality, and cost-effective cancer treatment strategies in the region.

### Declarations

All authors have no conflicts of interest to disclose.

### Author Contributions

Dr. Al Far, Dr. Abdulaziz, and Dr. Al Balushi represent different aspects of MCDA in the UAE, Bahrain, and Oman, respectively. All three shared their insights and contributed to the survey. After receiving the draft, most of them acknowledged it; however, only Dr. Sara updated the section related to Oman.

## Supplementary Material

Online Supplementary Material
