# Supplementary material for: Optimizing Oncology Generic Medication Selection in the Gulf Region: Expert Consensus and MCDA Tool Development
Source: J Health Econ Outcomes Res. 2025 Jul 18;12(2):21–6. doi: 10.36469/001c.140955 (PMC12276732; doi:10.36469/001c.140955)
Supplement: Online Supplementary Material [file jheor_2025_12_2_140955_294737.pdf]

Journal of Health Economics and Outcomes Research

Online Supplementary Material

Optimizing Oncology Generic Medication Selection in the Gulf Region: Expert Consensus and Multicriteria Decision Analysis Tool Development. *JHEOR*. 2025;12(2):21-26. doi:10.36469/jheor.2025.140955

Figure S1: Agenda of the Meeting

This supplementary material has been provided by the authors to give readers additional information about their work.

This is an open-access article distributed under the terms of the Creative Commons Attribution 4.0 International License (CCBY-4.0). View this license's legal deed at http://creativecommons.org/licenses/by/4.0 and legal code at http://creativecommons.org/licenses/by/4.0/legalcode for more information.

Figure S1. Agenda of the Meeting

Chairman : Dr. Anas Hamad

Time	Agenda
18:30 -18:35	• Welcome and introduction<br>• Review of meeting's purpose, objectives, and agenda
18:35 – 18:45	• Hikma Oncology portfolio
18:45 – 19:00	**Speaker: Dr. Mohammed AlFar**<br>• **Brands vs. high quality generics medications: Are patient outcomes affected? evidence from pharmacoepidemiologic studies.**
19:00 – 19:25	**Discussion: ALL**<br>• What are your experiences with prescribing high quality generic medications for oncology patients in the lower GCC region?<br>• What is the role of high-quality generics in cost containment?<br>• In your opinion, are lower GCC patients generally receptive to the use of generic medications for their oncology treatments? How do you manage any feedback they might have?<br>• What are the challenges you face in terms of availability and access to medications which improved by high-quality generics?
19:25 – 19:40	**Speaker: Dr. Sara Al Balushi**<br>• **Pharmacoeconomically considerations in formulary addition of oncology medications – challenges & opportunities**
19:40 – 20:05	**Discussion: ALL**<br>• How do you evaluate the cost-effectiveness of new oncology medications when considering formulary addition?<br>• What is the process for assessing the budget impact of adding a new oncology drug to the formulary, and what factors are most critical?<br>• How do you involve key stakeholders (physicians, payers, patients) in the decision-making process for formulary additions?
20:05 - 20:15	• Coffee Break
20:15 - 20:30	**Speaker: Dr. Omar Sulaiman**<br>• **Multi criteria decision analysis for off - patent cancer medications**
20:30 – 20:55	**Discussion: ALL**<br>• Most essential Quality measures for assessing off - patent oncology medications:<br>**Manufacturing quality - Cost - Supply reliability - Use in reference countries - Regulatory aspects - Provision of pharmacovigilance services - Extra services**<br>• What criteria do you consider most important in the multi-criteria decision analysis for off-patent cancer medications?<br>• How does the outcome of your multi-criteria decision analysis influence clinical practice and treatment protocols in your institution?<br>• How do you balance cost savings from off-patent medications with potential differences in efficacy or safety compared to branded alternatives? (Insurance Parameter)<br>• What are your recommendations for pharma companies for promoting health care education in GCC?
20:55 – 21:00	• Wrap-up and next steps
21:00	• Dinner

Abbreviation: GCC, Gulf Cooperation Council.
